# Monte Carlo simulation of photons backscattering from various thicknesses of lead layered over concrete for energies 0.25–20 MeV using FLUKA code

**DOI:** 10.1038/s41598-021-96026-y

**Published:** 2021-09-15

**Authors:** Ihsan A. M. Al-Affan, Mohammad A. Z. Qutub, Richard P. Hugtenburg

**Affiliations:** 1 Swansea University, Swansea, SA2 8PP UK; 2 Department of Physics, Umm Al-Qura University, Makkah, Saudi Arabia; 3 Department of Medical Physics and Clinical Engineering, Singleton Hospital, Swansea, SA2 8QA UK

**Keywords:** Radiotherapy, Applied physics

## Abstract

There is an increased interest in determining the photon reflection coefficient for layered systems consisting of lead (Pb) and concrete. The generation of accurate reflection coefficient data has implications for many fields, especially radiation protection, industry, and radiotherapy room design. Therefore, this study aims to calculate the reflection coefficients of photons for various lead thicknesses covering the concrete. This new data for lead, layered over concrete, supports various applications, such as an improved design of the mazes used for radiotherapy rooms, which helps to reduce cost and space requirements. The FLUKA Monte Carlo code was used to calculate photon reflection coefficients for a concrete wall with different energies. The reflection coefficient was also calculated for a concrete wall covered by varying thicknesses of lead to study the effect of lining this metal on the concrete wall. The concrete's reflection coefficient data were compared to internationally published data and showed that Monte Carlo calculations differed significantly from some of the extrapolated data. The absorbed dose of backscattered photons for various thicknesses of lead covering the ordinary concrete has been tabulated as a function of the reflection angle. Also, the reflection coefficient as a function of the Pb thicknesses covering the ordinary concrete has been figured to study the dose reduction factor. The generation of accurate data for reflection coefficients is vital for many fields, especially for radiation protection and radiotherapy room design. The new data have been presented for lead layered over concrete in various applications, such as an improvement in the design of the mazes used for radiotherapy rooms, thereby reducing the cost and space requirements. In addition, the Monte Carlo method enables calculating the energy distribution of reflected photons, and these were shown for a range of angles.

## Introduction

Data for backscattered photons from various materials are essential for shielding purposes, the quality of which has implications for many fields, especially radiation protection in industry and radiotherapy room design. Recent studies by Al-Affan et al.^1,2^ found a novel technique that can reduce the dose of backscattered photons at the radiotherapy room's maze entrance. This technique is based on using a few millimetres of lead to cover the concrete walls of the maze of a radiotherapy room. Dose rates of backscattering photons from radiotherapy rooms walls, floors and passageways can be equal or even exceed the dose rate of transmission photons through the room walls^3–6^.

Therefore, a knowledge of backscattering photons is important to reduce the radiation risks of passers-by and staff working near the treatment room. The concept of backscattering photons can be defined in terms of the Latin word ‘albedo’; that is, whiteness^7,8^. This term is expressed in a radiation field as the reflected photons' ratio from the material surface to those incidents on that surface. The albedo concept may also be termed as a reflection coefficient (RC). The reflected radiation takes into account photons that are backscattered from the surface and the medium's various depths. The RC depends on the mean free paths of the photons below the reflector surface. A dose albedo is commonly used for practical purposes, defined as the fraction of the incident dose that the surface reflects at certain angles^9,10^.

The Compton equation and the Klein–Nishina formulae were the early studies for the backscattered radiation in 1929, which led to a general expression for scattering and collisions of cross-sections of photons and electrons^11,12^. Hayward and Hubble studied Cobalt-60 (Co60) photon energy distributions as a function of the backscattered angles in 1954. However, the early comprehensive study of backscattering was given by Hyodo^13^, where the method of a scintillation spectrometer and the point gamma sources of Co^60^ and caesium-137 (Cs^137^) were used. Fujita et al. and Mizukami et al. arrived at an empirical formula of variation of photon backscattered values' number and energy by increasing the thickness of the scattered slab^14,15^. Their work proved that more than two mean free paths of the radiation source are enough to make the reflected material as infinite thickness.

Photon backscattering has already been the subject of numerous measurements and Monte Carlo calculations^16–22^. These studies concern the backscattering of various photon energies with different methods show almost similar results. However, the differential dose of backscattered photons for concrete at various angles, for bremsstrahlung and mono-energetic photons, have been tabulated in NCRP^23^. These values of the photon reflection coefficient are based on the evaluation of data from NCRP^23^, and Lo^24^. Furthermore, in the NCRP^23^, the reported uncertainty of the reflection coefficient values was on the order of ± 50% due to both the interpolations and the calculations.

In this research article, because ordinary concrete has a wide application as a radiation shielding material, simulations with FLUKA MC code was used to establish the possibility of introducing the ordinary concrete dose of backscattered photons for different mono-energetic incident photons. Then, the dose of backscattered photons of ordinary concrete covered with various thicknesses of lead is simulated. The photon beams would normally be incident on the target, and the energy range is between 250 keV to several MeV at various angles of the reflected beam. The relationship between the reflection coefficient (RC) and the thickness of the lead covering the ordinary concrete has been studied. The aim is to investigate the influence of various thicknesses of lead covering ordinary concrete. Also, the optimised thickness of lead for various incident photon energies has been calculated, which gives a maximum reduction of the backscattered photons dose. This research has produced detailed calculations for the backscattered dose over a wide range of angles. The dosemeter in this work is thin enough to minimally perturb the photons and thick enough to offer electronic equilibrium. In previous work, an average dose was calculated with a relatively larger dosemeter. Furthermore, the reflection coefficient was not calculated or measured before for a multi layered materials.

## Methods

The FLUKA Monte Carlo code, installed on a Linux Ubuntu operating system, an Intel CORE i7 desktop computer, and a High-Performance Computer (HPC Wales) were used to carry out the simulations. A code input file involves the radiation source and its energy, the beam's position, the materials, geometry, the number of primary photons, and their properties. A region that holds the whole system of radiation source and geometry is known as ‘the void’ surrounded by a ‘black-hole’ region. All the radiations that reach the black-hole would disappear because it has an infinite absorption cross-section. The geometry used in the simulations consists of different energy sources, water dosimeters and reflected materials. The photon energy cut-off was set to 1 keV, and the electron kinetic energy cut-off was set to 100 keV. Rayleigh scattering was taken into account^25,26^.

Flair is an integrated development environment for FLUKA; it uses a friendly interface to facilitate editing input files and imaging the output files^26,27^. A card is a keyword followed by a list of arguments. Below is a brief description of some of the most fundamental cards that are used in this work:

Primary Cards involve BEAM and BEAMPOS cards. A Beam Card defines the characteristics of a radiation beam with an arbitrary distribution of energy. BEAMPOS defines the position and direction of the radiation beam.

Geometry Cards: these cards allow the user to define the complex geometries that can be shown by graphical tools. There are multiple card options under the geometry elements, such as rectangular parallelepipeds (RPP) that are considered as a region. Then each region needs to assign its materials.

Media Cards: these cards are used to define different materials such as concrete for walls, water for the target and the dosimeters and air for the environment, etc.

Scoring Cards: there are several cards to score particles that pass through the dosimeters. The details of all cards above are discussed in the FLUKA manual^28^.

### Dose calculations $\left({\mathrm{D}}_{\mathrm{o}}\right)$ for various energies of incident photon beam at reflected materials

**Figure 1:**
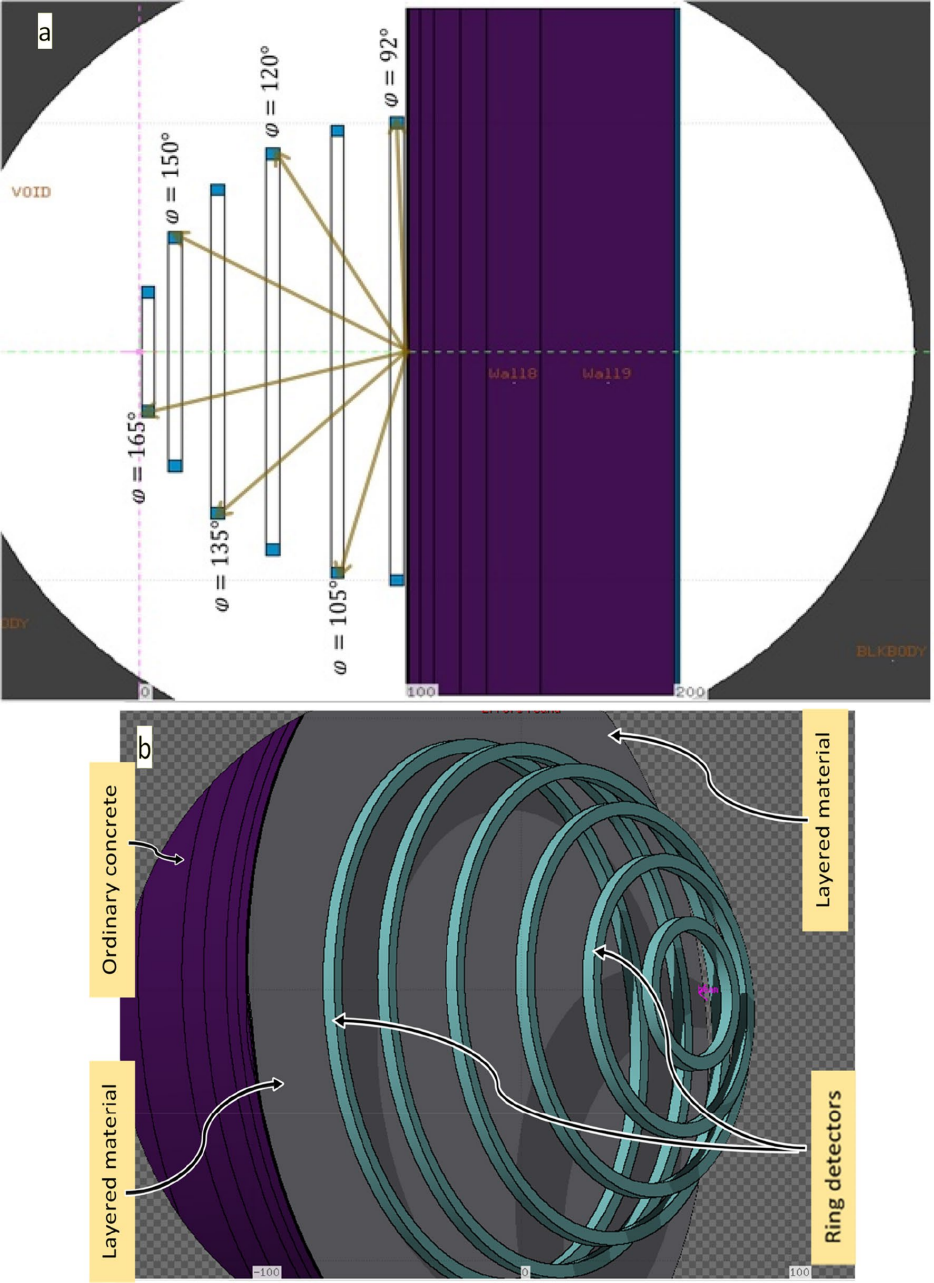
FLUKA Monte Carlo simulation of the ring dosimeters to calculate the dose of backscattered photons at reflection angles with respect to the incident trajectory, normal to the surface (**a**: is 2D and **b**: 3D image).

The FLUKA code was used to simulate the source that is a conically diverged photon beam with a 5.65 cm radius at the reflected material surface, giving an equivalent area of 10 × 10 cm^2^ field size (Fig. 1). The photon source was fixed at 100 cm from the surface of the rectangular parallelepiped water phantom. Inside the phantom, several dosimeters were simulated at various depths along the central axis. The dosimeters were rectangular and made of water, which is a suitable tissue equivalent material. The photons dose was calculated for those dosimeters inside the phantom. The maximum dose was obtained and considered as the incident dose (D_o_). The incident photons had energies of (0.25, 0.5, 0.662, 1, 1.25, 3, 7, 10, 15, and 20 MeV) to study several components of the X-ray spectrum usually present in the primary beam (of energies up to 20 MeV). The high energy is used for applications on big patients where the cancer is deep in the body.

### Reflection coefficients $\left(\mathbf{R}\mathbf{C}\right)$ of photon beams

The entire geometry of backscattered photons simulation was surrounded by a large sphere of a void of 1000 cm in a radius of vacuum, and this was surrounded by a larger sphere of a black-hole of 10,000 cm in radius^25^. The main reason for using vacuum was to avoid photon scattering in the air, which contaminates the result. The irradiations were carried out for a range of photon energies. For each photon energy, the FLUKA code was run for five cycles to determine the results' statistical fluctuation. Moreover, 70–230 million photon histories were generated for each simulation to get a statistical uncertainty of better than 4% (95% confidence limit). The computation time of the doses was between 60 and 140 h for five cycles for all situations.

To enhance the dosimeter efficiency and reduce the computation time, ring dosimeters made of water, were placed at 1 m in the radius of a vacuum semi-spherical object. The source of the incident photon beam was placed on the top of the sphere surface, the reflected materials were in the sphere center, and the ring dosimeters positioned at the sphere surface regarding its reflection angles $\left({\upvarphi }\right)$ as shown in Fig. 1. The ring dosimeters were 1 cm thick (between the inner and outer circles) and 1 cm in height. This size of dosimeters is capable of inducing electronic equilibrium and reduces the photons perturbation. The reflection angles with respect to the incident trajectory, normal to the surface, were taken at $92^\circ , 105^\circ , 120^\circ , 135^\circ , 150^\circ ,\, \mathrm{and}\,165^\circ$ as shown in Fig. 1. Using a simulated water dosimeter because water is a tissue equivalent material that directly compared with published results, including measurements.

The reflection coefficient $\left(\mathrm{RC}\right)$ can be calculated by the following relationship:

1 $$\mathrm{RC}=\frac{{D}_{\varphi }}{{D}_{o}}$$

where $\left({\mathrm{D}}_{\mathrm{o}}\right)$ is the incident dose and $\left({\mathrm{D}}_{{\upvarphi }}\right)$ is the absorbed dose of backscattered photons. The dose can be calculated from photon energy (E), where the FLUKA code gives the photon energy (E) deposited in the GeV unit^25^. Therefore, the dose can be obtained by converting the energy to joules and dividing by the mass of the dosimeter. FLUKA normalises its dose per incident particle (in this case, it is a photon)^25^.

Firstly, the RC of an ordinary concrete wall was calculated for the mentioned energies in “Dose calculations D_0_ for various energies of incident photon beam at reflected materials” section. The thickness of the reflected material was 100 cm. Then the ordinary concrete wall was lined with different thicknesses of lead (Pb). The ordinary concrete composition and density have been taken from NCRP^23^. The relationship between the RC and the angles for the various energies is plotted. Also, the relationship of RC values as a function of the lead's lining thickness has been illustrated.

## Results and discussion

### RC of an ordinary concrete

The reflection coefficient (RC) of an ordinary concrete was calculated using Eq. (1) and is shown in Table 1.

**Table 1:** The reflection coefficient of photons for ordinary concrete as a function of reflected angles calculated using the FLUKA code (wall-reflection coefficient).

Energy (MeV)	Angle of reflection or scatter (degrees) from surface of concrete					
	92	105	120	135	150	165
0.25	1.21	10.49	18.02	23.01	26.29	28.03
0.5	0.80	7.24	12.11	15.03	16.74	17.54
0.662	0.66	5.99	9.95	12.29	13.56	14.12
1	0.45	4.15	6.83	8.33	9.14	9.45
1.25	0.37	3.47	5.68	6.91	7.57	7.82
2	0.25	2.43	4.00	4.89	5.36	5.55
3	0.19	1.92	3.24	4.02	4.49	4.65
5	0.14	1.58	2.79	3.58	4.04	4.23
7	0.11	1.43	2.62	3.40	3.85	4.06
10	0.09	1.29	2.45	3.23	3.69	3.88
20	0.07	0.98	1.99	2.70	3.11	3.32

Multiply each table entry by 10^–3^ (e.g., the entry 5.39 means 5.39 × 10^–3^) with electrons cut-off = 100 keV and photons cut-off = 1 keV.

The statistical uncertainties are within ± 4% (95% confidence limit).

It can be seen from Table 1 that the reflection coefficient (RC) of an ordinary concrete wall increases with the increase of the backscattered angle for all photon energies. However, with increasing photon energy, the RC decreases as most photons would scatter in the forward direction.

**Figure 2:**
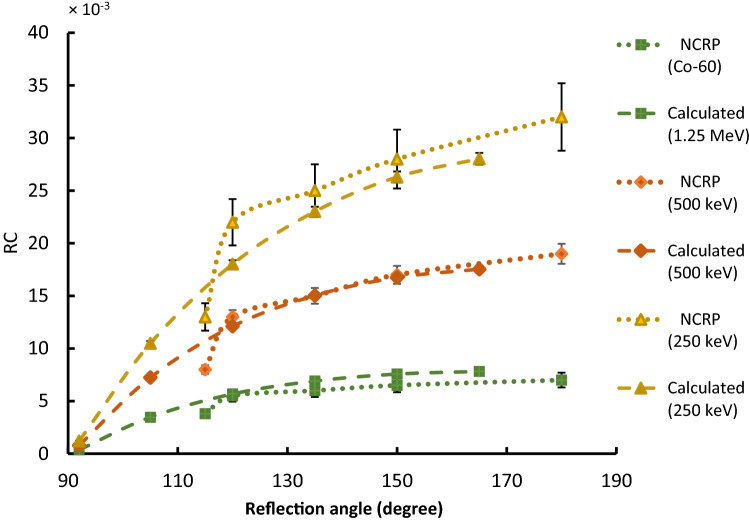
Comparisons between the backscattered photons of ordinary concrete of NCRP data and FLUKA calculations as a function of reflection angles (with respect to the incident trajectory, normal to the surface).

The available published data of backscattered photons of ordinary concrete shown in Lo^24^, and NCRP^23^ have significant uncertainties up to ± 50%. The large uncertainties were due to the calculations and the interpolations. The excellent agreement with the reflection coefficient (RC) of ordinary concrete using the FLUKA Monte Carlo code is within 10%, as shown in Fig. 2. However, the uncertainty of ordinary concrete of backscattered photons using the FLUKA calculations are accurate and less than 4% (95% confidence limit).

### RC of an ordinary concrete lined by various thicknesses of lead

The reflection coefficient (RC) for various thicknesses of lead (Pb) layered over ordinary concrete is calculated using Eq. (1). The thickness of ordinary concrete was 100 cm, while the lead thicknesses were 0.2 mm, 0.5 mm, 1 mm, 2 mm, 3 mm, 4 mm, 6 mm, 1 cm, and 2 cm. The results are represented in Table 2.

**Table 2:** The reflection coefficient of photons for various Pb thickness layers layered over ordinary concrete as a function of reflected angles calculated using the FLUKA code (wall-reflection coefficient).

Energy (MeV)	Angle of reflection or scatter (degrees) from surface of concrete					
	92	105	120	135	150	165
**0.2 mm Pb**						
0.25	0.47	4.37	10.02	14.79	18.14	19.67
0.662	0.28	3.99	7.87	10.61	12.30	12.96
1.25	0.20	2.74	5.15	6.83	7.87	8.21
3	0.15	1.85	3.52	4.78	5.55	5.79
5	0.13	1.65	3.25	4.47	5.21	5.43
7	0.11	1.54	3.05	4.15	4.83	5.05
10	0.09	1.38	2.79	3.77	4.39	4.59
15	0.07	1.19	2.45	3.36	3.86	4.05
20	0.06	1.02	2.13	2.93	3.43	3.61
**0.5 mm Pb**						
0.25	0.33	2.40	5.38	8.32	10.47	11.42
0.662	0.20	2.42	5.27	7.40	8.80	9.33
1.25	0.15	2.00	3.99	5.46	6.37	6.74
3	0.16	1.87	3.73	5.35	6.40	6.78
5	0.15	1.94	4.12	6.13	7.48	7.94
7	0.14	1.88	4.08	6.05	7.40	7.82
10	0.12	1.70	3.71	5.47	6.58	6.95
15	0.09	1.44	3.13	4.51	5.41	5.61
20	0.07	1.22	2.64	3.75	4.46	4.70
**1 mm Pb**						
0.25	0.29	2.02	4.07	6.02	7.44	8.04
0.5	0.16	1.38	3.17	4.77	5.88	6.34
0.662	0.16	1.47	3.31	4.79	5.78	6.07
1	0.08	0.89	1.89	2.65	3.16	3.31
1.25	0.13	1.42	2.95	4.10	4.86	5.09
2	0.15	1.58	3.10	4.34	5.14	5.37
3	0.17	1.79	3.59	5.15	6.24	6.63
5	0.19	2.18	4.61	6.98	8.63	9.24
7	0.19	2.34	5.15	7.92	9.98	10.69
10	0.18	2.30	5.13	7.99	10.02	10.65
15	0.14	1.96	4.43	6.75	8.42	8.98
20	0.11	1.65	3.69	5.53	6.82	7.20
**2 mm Pb**						
0.25	0.29	1.99	3.89	5.69	6.92	7.40
0.5	0.14	1.07	2.14	3.14	3.86	4.08
0.662	0.13	1.05	2.12	3.07	3.70	3.91
1	0.07	0.62	1.27	1.81	2.16	2.25
1.25	0.11	1.04	2.11	2.97	3.55	3.65
2	0.14	1.41	2.71	3.79	4.49	4.70
3	0.17	1.82	3.52	4.97	6.02	6.33
5	0.20	2.39	4.87	7.23	8.94	9.51
7	0.22	2.76	5.77	8.77	11.01	11.65
10	0.24	3.01	6.40	9.83	12.37	13.46
15	0.22	2.88	6.19	9.57	12.12	13.19
20	0.18	2.49	5.36	8.24	10.40	11.25
**3 mm Pb**						
0.25	0.29	1.98	3.90	5.66	6.91	7.42
0.5	0.14	1.05	1.99	2.87	3.50	3.68
0.662	0.13	1.00	1.86	2.65	3.19	3.35
1	0.07	0.57	1.08	1.52	1.82	1.89
1.25	0.11	0.95	1.81	2.59	3.06	3.20
2	0.14	1.39	2.60	3.63	4.31	4.52
3	0.17	1.86	3.57	5.05	6.07	6.42
5	0.21	2.55	5.11	7.48	9.18	9.75
7	0.23	3.04	6.13	9.18	11.36	12.12
10	0.26	3.42	6.97	10.47	13.19	14.05
15	0.26	3.52	7.09	10.71	13.48	14.52
20	0.22	3.18	6.44	9.68	12.13	13.17
**4 mm Pb**						
0.25	0.29	1.99	3.90	5.66	6.90	7.42
0.5	0.14	1.04	1.96	2.81	3.41	3.63
0.662	0.13	0.97	1.80	2.55	3.05	3.20
1	0.07	0.55	1.02	1.44	1.70	1.77
1.25	0.11	0.91	1.72	2.41	2.91	2.99
2	0.14	1.39	2.58	3.61	4.29	4.48
3	0.17	1.91	3.64	5.16	6.19	6.55
5	0.21	2.67	5.32	7.73	9.50	10.09
7	0.24	3.23	6.46	9.51	11.82	12.56
10	0.27	3.72	7.40	11.04	13.67	14.66
15	0.27	3.98	7.78	11.42	14.24	15.40
20	0.24	3.73	7.21	10.53	13.18	14.21
**6 mm Pb**						
0.25	0.29	1.99	3.90	5.65	6.91	7.41
0.662	0.13	0.98	1.79	2.50	2.98	3.15
1.25	0.11	0.91	1.67	2.35	2.80	2.93
3	0.17	1.96	3.81	5.42	6.53	6.85
5	0.21	2.83	5.65	8.19	9.96	10.66
7	0.25	3.48	6.93	10.17	12.44	13.24
10	0.28	4.13	8.12	11.86	14.66	15.53
15	0.28	4.58	8.85	12.67	15.61	16.59
20	0.26	4.51	8.43	11.95	14.62	15.64
**1 cm Pb**						
0.25	0.29	1.99	3.89	5.66	6.93	7.42
0.662	0.13	0.97	1.78	2.49	3.01	3.16
1.25	0.11	0.91	1.68	2.35	2.84	2.94
3	0.17	2.02	4.00	5.76	6.93	7.37
5	0.22	2.96	6.03	8.76	10.70	11.45
7	0.25	3.72	7.50	10.96	13.36	14.22
10	0.29	4.55	8.92	12.99	15.87	16.83
15	0.30	5.24	9.96	14.14	17.15	18.40
20	0.28	5.27	9.88	13.67	16.49	17.59
**2 cm Pb**						
0.25	0.29	1.99	3.89	5.67	6.91	7.37
0.662	0.13	0.98	1.77	2.50	2.98	3.18
1.25	0.11	0.91	1.68	2.36	2.87	2.97
3	0.17	2.05	4.12	5.97	7.30	7.78
5	0.22	3.04	6.31	9.24	11.32	12.14
7	0.26	3.85	7.96	11.63	14.31	15.23
10	0.29	4.78	9.64	13.97	17.08	18.26
15	0.31	5.58	10.94	15.56	18.84	20.11
20	0.29	5.70	11.12	15.42	18.56	19.76

Multiply each table entry by 10^–3^ (e.g., the entry 5.39 means 5.39 × 10^–3^) with electrons cut-off = 100 keV and photons cut-off = 1 keV.

The statistical uncertainties are within ± 4% (95% confidence limit).

**Figure 3:**
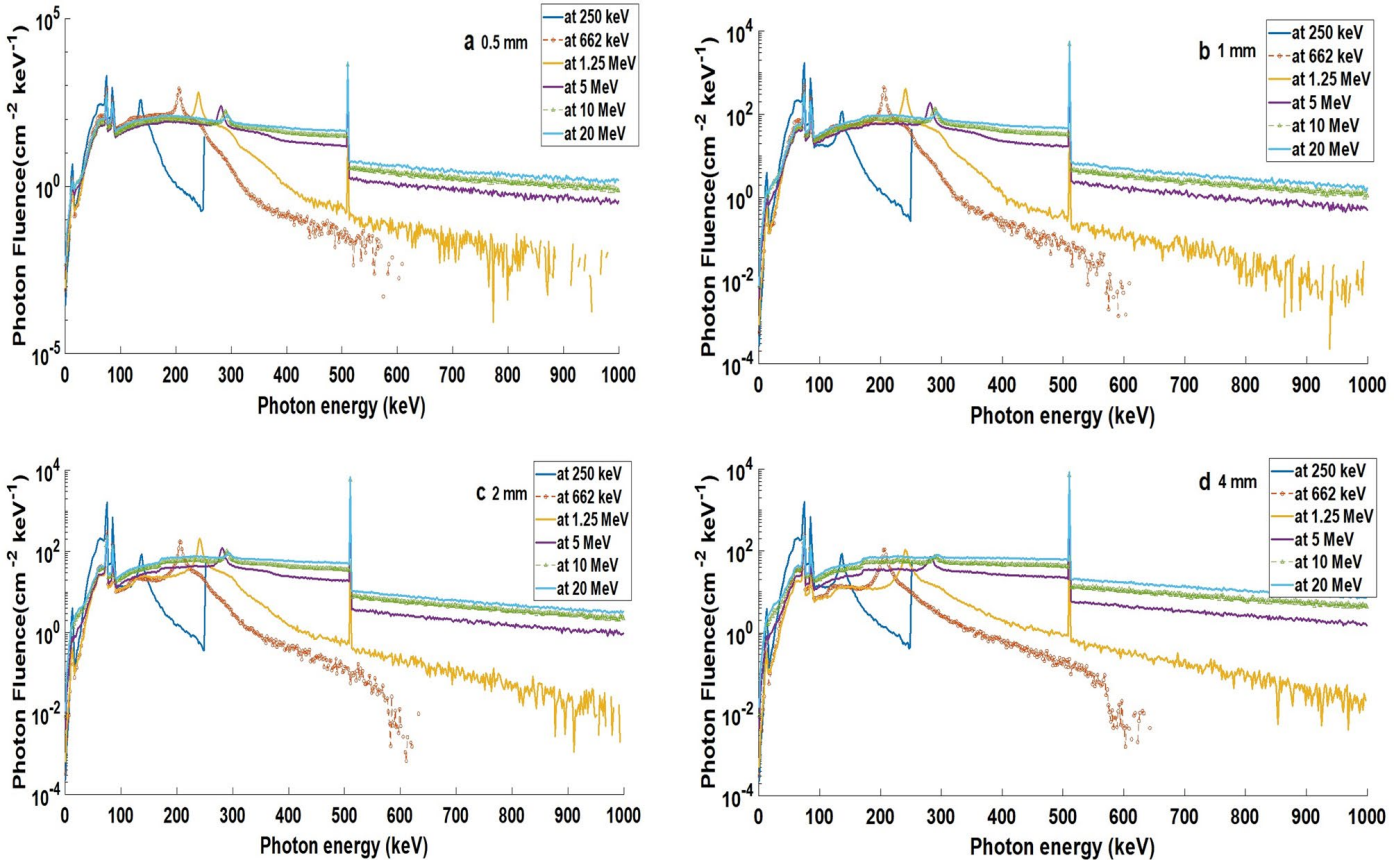
The fluence spectra of backscattered photons of various energy for (**a**): 0.5 mm, (**b**): 1 mm, (**c**): 2 mm, and (**d**): 4 mm Pb layered over the ordinary concrete at a reflection angle of 135°.

Table 2 shows that the 2 mm optimised thickness of lead (Pb) layered over concrete is enough to cause maximum reduction of the backscattered photons. Figure 3 shows the backscattered photons spectra for 0.5 mm, 1 mm, 2 mm, and 4 mm Pb layered over the ordinary concrete at a reflection angle of 135°. These spectra can be obtained by a flair interface USRTRACK card that is defined as a dosimeter for a track-length fluence estimator^25^.

**Figure 4:**
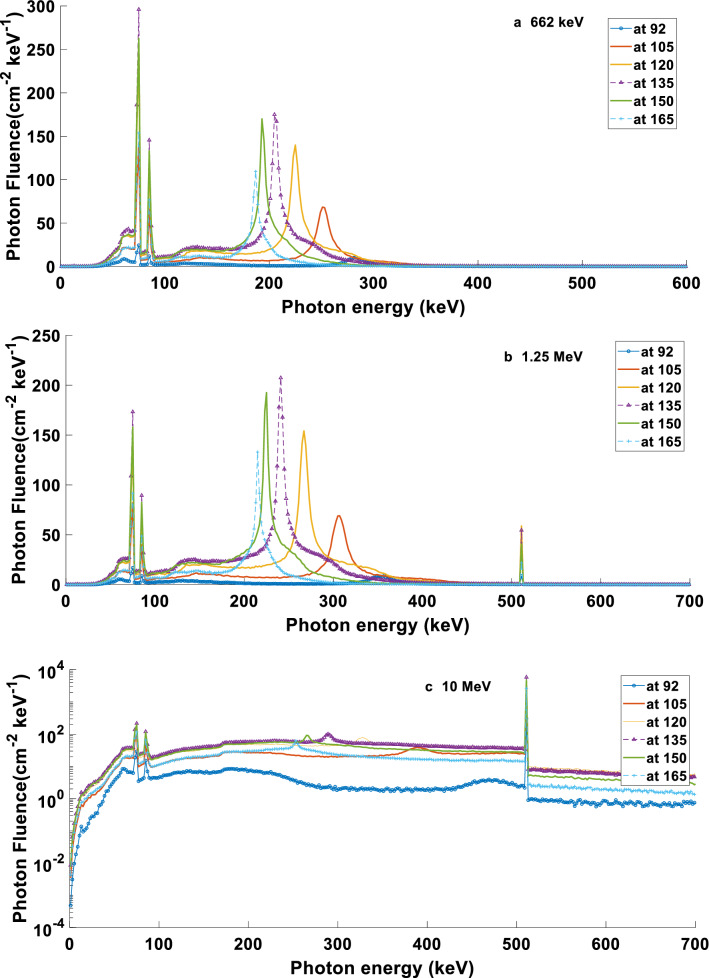
The fluence spectra of backscattered photons at various reflection angles of 2 mm Pb layered over ordinary concrete and incident photon energies of (**a**): 662 keV, (**b**): 1.25 MeV, and (**c**): 10 MeV.

Table 2 shows that regardless of the thicknesses of lead covering the concrete, the RC increases with the backscattered angle. However, Fig. 4 shows that up to a reflected angle of 135° (i.e. 92°, 105°, 120°, and 135°), the amplitude of backscattered photons spectra increases while the photon energy decreases. Then, above these angles (i.e. 150° and 165°), the amplitude decreases, and its energy decreases.

**Figure 5:**
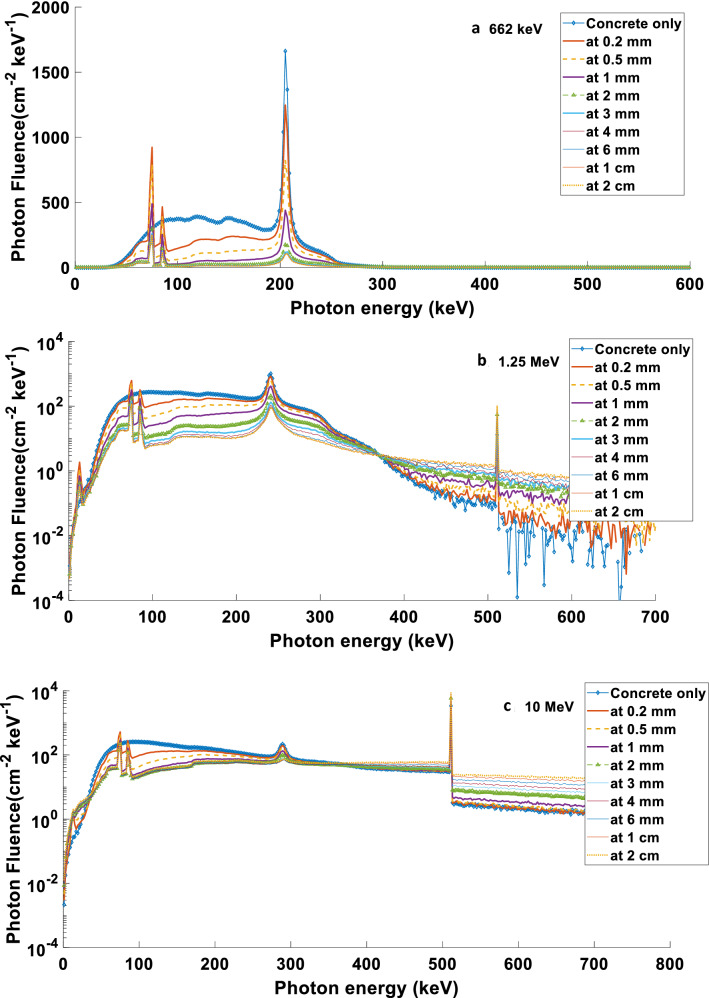
The fluence spectra of backscattered photons with the various thicknesses of Pb layered over ordinary concrete at reflection angle of 135° and incident photon energies of (**a**): 662 keV, (**b**): 1.25 MeV, and (**c**): 10 MeV.

Because lead is considered a material with a high atomic number (Z), there are two peaks in constant positions, regardless of the incident energy. These peaks are the orbital (K) radiation at 79 keV and the Compton peak, followed at high energies, as shown in Fig. 3. This figure shows the backscattered photons spectra of various incident energies for specific thicknesses of Pb that covered the ordinary concrete. These peaks appear regardless of Pb thicknesses layered over the ordinary concrete, as shown in Fig. 5.

When photon energy increases above 1.25 MeV, the RC increases as some photons with energies above 1.02 MeV start the pair production effect when interacting with high Z materials. Hence the electron–positron product would annihilate to produce two photons with energy of 511 keV in opposite directions. That means one of their photons would be in the directions of backscattered photons. This phenomenon would increase with photon energy until the pair production would be dominant when the photon reaches an energy of about 5 MeV and above. The dominant peak of 511 keV is illustrated in Figs. 3 and 5.

**Figure 6:**
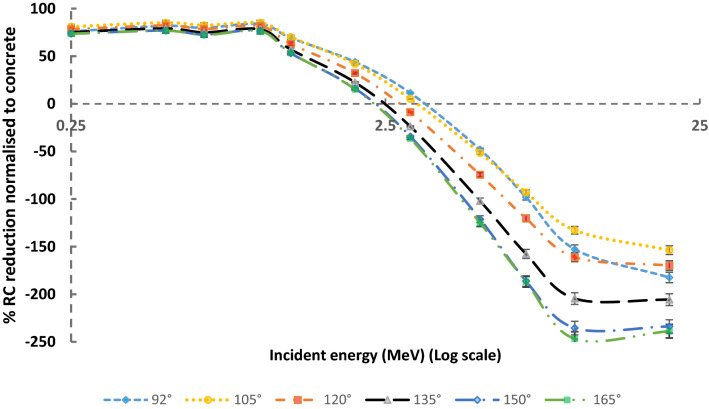
The percentage reduction factors of reflection coefficient (% RC) normalised to ordinary concrete for 2 mm Pb covering the ordinary concrete as a function of incident photons energy for variance reflection angles.

**Figure 7:**
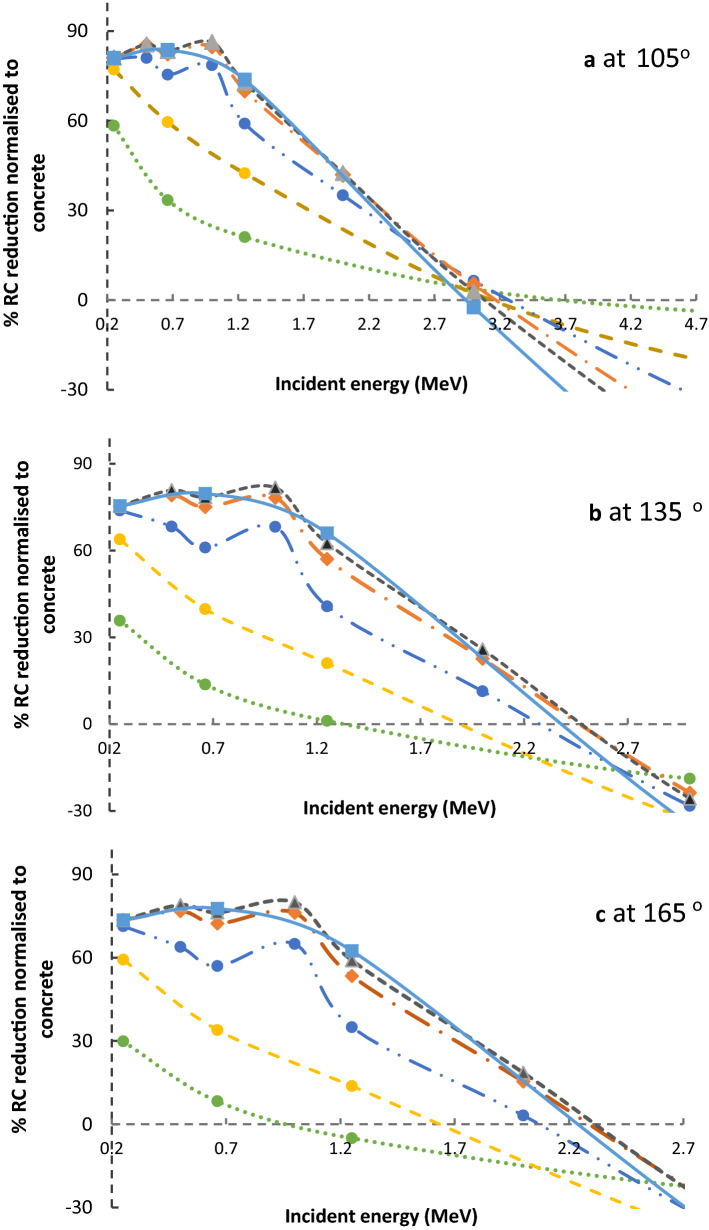
The percentage reduction factors of reflection coefficient (% RC) normalised to ordinary concrete for various thickness of lead covering the ordinary concrete as a function of incident photons energy. (**a**) at reflection angles of 105°, (**b**) at reflection angles of 135°, and (**c**) at reflection angles of 165°.  represents 0.2 mm Pb,  represents 0.5 mm Pb,  represents 1 mm Pb,  represents 2 mm Pb,  represents 3 mm Pb, and  represents 6 mm Pb.

The 2 mm of lead covering the concrete is an optimised thickness to reduce the dose for low energy incident photons, as shown in Figs. 6 and 7. These figures give the percentage reduction factors of RC normalised to ordinary concrete only with various incident photons' energies. The negative values indicate the increase of RC. These figures show that either 2 mm of lead covering the concrete is an optimised thickness, but for no more than 3 MeV at a reflected angle of 105°, 2.6 MeV at a reflected angle of 135°, and 2.3 MeV at a reflected angle of 165°. This thickness is equal to one or two mean free paths of most backscattered photons; hence, they absorb them by the photoelectric effect.

## Conclusions

The data of backscattered photons is used nowadays for various applications based on empirical formulae and extrapolations of several experiments carried out in the last 50 years. However, NCRP 151^23^ confirms that uncertainties in the results are of the order of ± 50% due to generation of both the methods used in the calculations and the extrapolations. Therefore, the reflection coefficient would improve the necessary data for international guidelines on the design of high energy X-ray installations, industry, radiation protection, and in the design of the beam stoppage of the tomotherapy linear accelerator. The material of the stoppage may vary from lead to iron depending on the manufacturer and the beam energy. Furthermore, there has been an interest in layered materials of the stoppage, for which no such data currently exists. Moreover, this research maintained a low statistical uncertainty of less than 4% (95% confidence limit) to achieve a practical accuracy for future comparison with calculations and measurements.

As in this work, the basic data of high energy X-ray installations have been produced and improved for international guidelines on a radiotherapy department's design. The results here, are in close agreement qualitatively with previously published results, particularly Al-Affan et al.^1,2^ relating to dose reduction when the few mm of lead is added to the concrete wall at the maze entrance. However, the present study aimed to calculate the reflection coefficient of photons with various energies for multi-layer materials. This new work shows that 2 mm of lead covering the concrete is suitable for reducing the photon dose in the incident energy range below 2.5 MeV, for various applications. This would exclude the use of lead lining for very high energy photons; however, the bremsstrahlung spectrum of LINAC X-rays consists of a broad range of photons and the technique may still be suitable for the majority of clinical installations, i.e. 6 and 10 MV nominal energies.
